# ASGR1 deficiency improves atherosclerosis but alters liver metabolism in ApoE^-/-^ mice

**DOI:** 10.1186/s12933-024-02507-5

**Published:** 2024-11-30

**Authors:** Monika Svecla, Annalisa Moregola, Lorenzo Da Dalt, Jasmine Nour, Andrea Baragetti, Patrizia Uboldi, Alessandra Idini, Manfred Wuhrer, Giangiacomo Beretta, David Falck, Fabrizia Bonacina, Giuseppe Danilo Norata

**Affiliations:** 1 Department of Pharmacological and Biomolecular Sciences, Università degli Studi di Milano, Milan, Italy; 2 Center for Proteomics and Metabolomics, Leiden University Medical Center, Leiden, The Netherlands; 3 Department of Environmental Science and Policy, Università degli Studi di Milano, Milan, Italy

**Keywords:** Atherosclerosis, Asialoglycoprotein receptor 1, Cholesterol, Plaque composition, Liver metabolism

## Abstract

**Abstract:**

The asialoglycoprotein receptor 1 (ASGR1), a multivalent carbohydrate-binding receptor that primarily is responsible for recognizing and eliminating circulating glycoproteins with exposed galactose (Gal) or *N*-acetylgalactosamine (GalNAc) as terminal glycan residues, has been implicated in modulating the lipid metabolism and reducing cardiovascular disease burden. In this study, we investigated the impact of ASGR1 deficiency (ASGR1^−/−)^ on atherosclerosis by evaluating its effects on plaque formation, lipid metabolism, circulating immunoinflammatory response, and circulating *N*-glycome under the hypercholesterolemic condition in ApoE-deficient mice. After 16 weeks of a western-type diet, ApoE^−/−^/ASGR1^−/−^ mice presented lower plasma cholesterol and triglyceride levels compared to ApoE^−/−^. This was associated with reduced atherosclerotic plaque area and necrotic core formation. Interestingly, ApoE^−/−^/ASGR1^−/−^ mice showed increased levels of circulating immune cells, increased AST/ALT ratio, and no changes in the *N*-glycome profile and liver morphology. The liver of ApoE^−/−^/ASGR1^−/−^ mice, however, presented alterations in the metabolism of lipids, xenobiotics, and bile secretion, indicating broader alterations in liver homeostasis beyond lipids. These data suggest that improvements in circulating lipid metabolism and atherosclerosis in ASGR1 deficiency is paralleled by a deterioration of liver injury. These findings point to the need for additional evaluation before considering ASGR1 as a pharmacological target for dyslipidemia and cardiovascular disorders.

**Graphical abstract:**

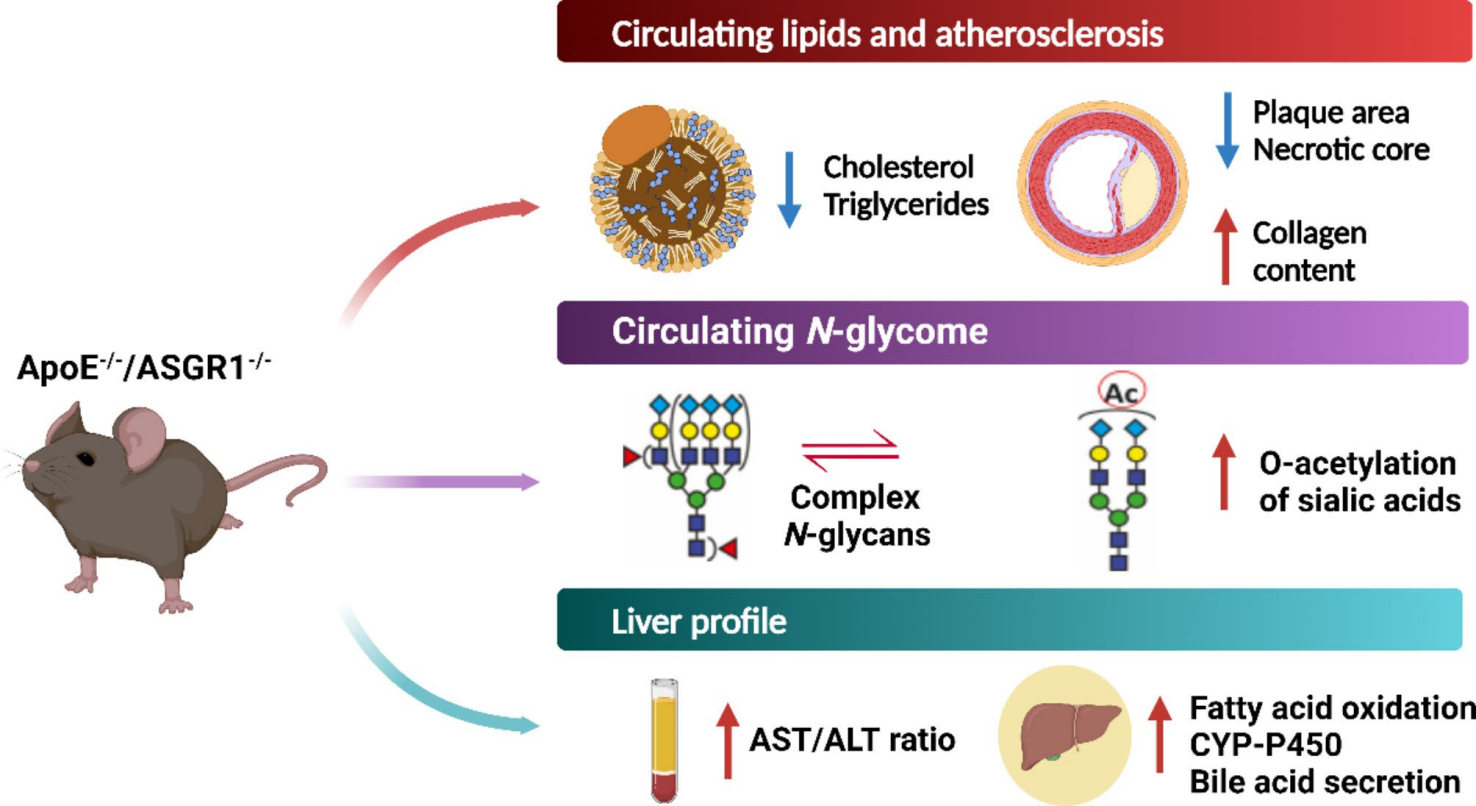

**Supplementary Information:**

The online version contains supplementary material available at 10.1186/s12933-024-02507-5.

## Introduction

Elevated plasma cholesterol levels are a prerequisite for the development of atherosclerosis [1] and despite the availability of several lipid lowering therapies with complementary mechanisms of action, there is still a lot of interest in understanding novel molecular mechanisms controlling lipids and lipoprotein metabolism [2]. Genome-wide association studies have identified over 200 loci, that influence the risk of atherosclerotic cardiovascular disease [3, 4] and, among them, asialoglycoprotein receptor 1 (ASGR1), the functional subunit of asialoglycoprotein receptor (ASGPR), emerged as a promising candidate affecting plasma cholesterol metabolism and cardiovascular disease (CVD) risk [5].

ASGR1 is a multivalent carbohydrate-binding receptor, expressed exclusively on the surface of hepatocytes [6]. The main function of ASGR1 is the recognition and the clearance of circulating desialylated glycoproteins, where sialic acid has been removed, and galactose (Gal) or *N*-acetylgalactosamine (GalNAc) are exposed as terminal glycan residues [7–9]. This binding and recognition are largely independent of the protein structure, thereby ASGR1 has a broad range of proteins that it can bind and remove from circulation. Indeed, recent studies of plasma proteomics associations with genetics in the UK Biobank revealed that ASGR1 is highly pleiotropic and is associated with at least 100 proteins [10]. The loss-of-function variants in the ASGR1 are associated with a significant reduction in cholesterol levels in non-HDL lipoproteins and a 34% reduction in a predicted cardiovascular disease (CVD) risk [5, 11]. Notably, in this study, the reduction observed in non-HDL cholesterol levels was partly explaining the protection from CVD disease supporting the possibility that ASGR1 could play an additional role in lipoprotein metabolism as well as in atherosclerosis. In vitro and in vivo studies showed that ASGR1 deficiency leads to reduced plasma cholesterol levels [12–14] via a mechanism involving the activation of liver X receptor (LXR) and AMP-activated protein kinase (AMPK). Increased LXR activity is known to enhance cholesterol efflux and excretion into the bile [12]. An independent study revealed that the decrease in lipid levels in ASGR1 deficiency is associated with the retention of insulin-induced gene 1 (INSIG1) and sterol regulatory element-binding protein (SREBP) in the endoplasmic reticulum, thus limiting the transcriptional activation of cholesterol synthesis [13].

These mechanisms, however, are not enough to explain the reduced risk of cardiovascular events observed in patients carrying loss of function mutations in the ASGR1 genes. Given the role of ASGR1 in modulating the recognition of desialylated glycoproteins, which is crucial during infection and inflammatory conditions [15], we investigated the effect of ASGR1 deficiency on the immunoinflammatory response observed during atherogenesis. To this aim, we have generated double-knockout mice lacking ASGR1 (ASGR1^−/−^) and ApoE^−/−^ and characterized the development of atherosclerosis. Our data show that under hypercholesterolemic conditions, ASGR1 deficiency results in improved plasma lipid profile and atherosclerotic plaque features by reducing necrotic core formation and promoting plaque stabilization through increased collagen content. Interestingly, the *N*-glycome in ASGR1 deficient mice is not affected during atherogenesis. Conversely, in the liver, ASGR1 deficiency enhances cytochrome P450 (CYP-P450) related protein expression which associates with altered lipid metabolism, bile acid production, and xenobiotic metabolism.

## Materials and methods

### Animals

ApoE^−/−^ and ASGR1^−/−^ mice (B6.129S4-Asgr1tm1Sau/SaubJxm) were purchased from Jackson Laboratory (Bar Harbor, ME, USA). Male and female, ApoE^−/−^, (C57BL/6J) were backcrossed for 10 generations with ASGR1^−/−^ mice (C57BL/6J and C57BL/6 N). Both male and female mice, were kept under controlled light/dark cycle (12 h of light / 12 h of dark) and temperature-controlled conditions (21 °C). At 8 weeks of age, mice were fed a Western-type diet (WTD, Envigo, Teklad Custom Diets, Cat#TD.88137), containing 42% of calories from fat and enriched with additional 0.2% cholesterol for 16 weeks. The procedures were performed conforming to the guidelines from the 2010/63/EU directive of the European Parliament on the protection of animals used for scientific purposes and were approved by the Ethical Committee of the University of Milan and the ISS instate of the Italian Ministry of Health (Approval number: Progetto di Ricerca 1171/2020).

### Lipid profile

Total plasma cholesterol and triglycerides were determined from frozen plasma using standard enzymatic procedures with the Cholesterol CP KIT (ABX Pentra, HORIBA Medical) or the triglyceride CP KIT (ABX Pentra, HORIBA Medical). Cholesterol and triglyceride concentrations were read by spectrophotometer at 490 nm (Bio-Rad iMark microplate reader). For lipid extraction, 10 mg of liver tissue were used. First, the tissue was homogenized with 1 mL of extracting solution (Methanol: Acetonitrile 1:1 (v/v)) using a tissue lyser at maximum power for 1 min. The homogenate was then incubated for 15 min at 37 °C with continuous shaking at 850 rpm, followed by centrifugation at 16,000x g for 10 min at 20 °C. The supernatant was transferred to a new tube and centrifuged again at 16,000x g for 3 min. The resulting solution was recovered and dried under nitrogen. The dried sample was reconstituted in 100 µL of MeOH/H2O (1:1 v/v), incubated for 10 min at 20 °C with agitation at 1300 rpm, sonicated for 10 min at room temperature, and centrifuged as before. Quantitative data were normalized to total liver protein content determined by the Lowry assay. Data are presented as ng/mg of liver protein.

### FPLC analysis of plasma lipoproteins and in vivo VLDL production assay

Size fractionation of plasma lipoproteins was performed with fast protein liquid chromatography, with a Superose 6 column (GE Healthcare, Chicago, IL, USA) coupled with NGCTM chromatography system FPLC (Bio-Rad laboratories Inc., Hercules, CA, USA) with an eluent solution containing 0.15 M NaCl pH 7.2 + 0.01% EDTA + 0.02% sodium azide. A plasma volume of 0.300 mL was loaded, with a flow rate of 0.25 mL/min. From each eluted fraction 0.5mL is collected and the concentration of cholesterol and triglycerides were measured as described previously [16]. For the in vivo hepatic VLDL production assay, mice were fasted for 4 h and then received an intraperitoneal injection of poloxamer 407 (1 mg/g mouse; Merck, Cat: #16758) to inhibit LPL activity. Blood was collected at the indicated time points and triglyceride levels were measured and presented as mmol/L and the secretion rate is presented as µmol/kg/h as previously described [17].

### Blood preparation for immunophenotyping

Immunophenotyping was performed on 50 µL of fresh blood. Initially, the blood was incubated with a specific antibody mixture at room temperature for 30 min. Afterward, red blood cells were fixed and lysed using Fix& lyse 1x solution (Thermofisher), followed by two washes with PBS containing 2% FBS and 2mM EDTA. Samples were then acquired on a BD LSRFortessa™ X-20 cytometer and analyzed by Novoexpress software (version 1.6.0, Agilent, Santa Clara, CA, USA) as previously described [18]. The antibodies used for staining are listed in Supp. Tab. S1.

### Plasma alanine aminotransferase (ALT) and aspartate aminotransferase (AST) measurements

Plasma levels of Alanine Aminotransferase (ALT) and Aspartate Aminotransferase (AST) were measured with commercially enzymatic kits and all the samples were run on an automatic analyzer (Randox, Crumlin, Ireland) as previously described [19]. The following method is indicated in the International Federation of Clinical Chemistry (IFCC) (for ALT: Cat#AL8006; R1 6 × 56 ml (L) and R2 6 × 20 ml (Mod. IFCC); for AST: Cat#AS8306; R1 4 × 20 ml (L) and R2 4 × 7 ml).

### Plasma *N*-glycan release and sialic acid derivatization

Plasma *N*-glycan release was performed with peptide-N-glycosidase F (PNGaseF, Roche, Mannheim, Germany). For denaturation 12 µL of 2% SDS were added to 6 µL of plasma and incubated for 10 min at 60 °C. Subsequently, 12.6 µL of the release mixture (6 µL 4% NP40, 6 µL 5× PBS, and 0.6 µL PNGase F) were added and the samples were incubated overnight at 37 °C. Sialic acid derivatization was performed as previously described [20]. In brief, from the released glycans 2 µL were added to 40 µL of ethyl esterification reagent (0.25 M EDC with 0.25 M HOBt in ethanol), after which the mixture was incubated for 1 h at 37 °C. This reaction introduced a mass difference between α2,3-linked sialic acids, losing water through lactonization, and α2,6-linked sialic acids, gaining C_2_H_4_ through esterification with ethanol [21]. Afterwards, 40 µL of acetonitrile were added and after 10 min of incubation, the purification was started. In-house assembled microtips used for cotton HILIC microtip purification were prepared as follows: 3 mm cotton thread (approximately 180 µg, Pipoos, Utrecht, Netherlands) were placed into a 50 µL tip (clear CO-RE tip without filter, Hamilton, Bonaduz, Switzerland) by using tweezers. Then, a porous polypropylene frit (DPX Technologies, Columbia GA, United States of America) was placed 18 mm above the tip opening. The cotton HILIC tips were three times pre-wetted with 40 µL of MQ water and then conditioned with three times 40 µL of 85% ACN. Subsequently, the sample was loaded by pipetting the ethyl-esterified sample 20 times up and down (40 µL per time). The HILIC tips were washed three times with 40 µL of 85% ACN containing 1% TFA, and three times with 40 µL of 85% ACN. The purified *N*-glycans were eluted in 20 µL of MQ water by pipetting five times. Next, 10 µL of purified sample was premixed with 5 µL of sDHB matrix (5 mg/mL in 99% ACN with 1 mM NaOH, Sigma-Aldrich, Steinheim, Germany) and 3 µL of the mixture was spotted onto a MALDI target plate (800/384 MTP AnchorChip, Bruker Daltonics, Bremen, Germany).

### MALDI-FT-ICR MS and glycan annotation

Samples were acquired in matrix-assisted laser desorption/ionization fourier transform ion cyclotron resonance mass spectrometry (MALDI-FT-ICR MS) as previous described [22]. Briefly, samples were processed using a 15 T solariX XR FT-ICR mass spectrometer equipped with a CombiSource and a ParaCell (Bruker Daltonics). The FT-ICR MS system was controlled by the ftmsControl software, equipped with a Smartbeam-II Laser System (Bruker Daltonics) that operates at 500 Hz. Each single spectrum was generated using 200 laser shots. The mass spectra were acquired from a single spot for the *m*/*z*-ranges: *m/z* 1000–5000. The data were obtained in serial mode and a single combined file was generated. The data were processed with DataAnalysis (ver. 5, Bruker Daltonics). The MALDI-FT-ICR MS file were divided into separate sum spectra for each sample (x, y files). For glycan annotation the mMass software was utilized and each of these spectra the calibration was performed using MassyTools. The Massy Tools output file was further used for analyte curation, with S/*N* ≥ 9, isotopic pattern quality ≤ 0.45 and mass accuracy between ± 20 ppm. The glycan compositions passing these criteria in 45% of the total number of samples were considered for further analysis. The peak areas of the curated glycans were calculated for each spectrum and relative abundances determined by total area normalization. To base the analysis on common structural features, rather than on individual glycans, the glycosylation traits were calculated. Glycans were abbreviated according to their monosaccharide composition (hexose = H; N-acetylhexosamine = N; fucose = F; α2-6-linked N-acetylneuraminic acid = E; α2-3-linked N-acetylneuraminic acid = L; N-glycolylneuraminic acid = Ge or Gl for α2-6- and α2-3-linked variants, respectively) and the glycan compositions are assigned as [M + Na]^+^.

### Liver proteomics and in-solution tryptic digestion

Livers (*n* = 4/group) were pooled up to 20 mg and lysed with Urea 8 M, Tris-HCl 0.1 M pH 8.5 in the presence of protease inhibitors at a ratio of 1:100 (Cell Signaling, Cat# 5872 S) for 60 min at 4 °C with constant shaking. Next, samples were centrifuged for 30 min at 14,000 g at 4 °C. The supernatant containing the proteins extracted was collected and quantified by Lowry protein assay. Next, samples were then dried completely using a vacuum concentrator at 45 °C for 45 min and later resuspended in 10 µl of water with the addition of 10 µl of Ammonium bicarbonate solution 50 mM (final pH 8.5). Proteins were reduced following incubation with DTT (final concentration 5 mM), for 30 min at 55 °C. Protein alkylation was then performed at room temperature, by incubating with iodoacetamide (final concentration 15 mM), for 30 min in the dark. Trypsin digestion (enzyme to protein ratio of 1:20), was performed overnight at 37 °C, and stopped by acidification trifluoroacetic acid, (final percentage: 1%).

### LC-MS/MS analysis and proteomics data processing

Samples were acquired in Dionex Ultimate 3000 nano-LC system (Sunnyvale CA, USA) connected to an orbitrap Fusion™ Tribrid™ Mass Spectrometer (Thermo Scientific, Bremen, Germany) equipped with a nanoelectrospray ion source. The peptide mixtures were pre-concentrated onto an Acclaim PepMap C18 5 μm, 100 Å, 100 μm ID x 2 cm (Thermo Scientific) and separated at 35 °C on an EASY-Spray PepMap RSLC C18 column: 3 μm, 100 Å, 75 μm ID x 25 cm (Thermo Scientific), using mobile phase A (0.1% aqueous formic acid) and mobile phase B (0.1% aqueous formic acid /acetonitrile (2:8)) at a flow rate: 300 nL/min. MS spectra were collected for the *m/z* ranges of 375–1500 Da, at 120.000 resolution, in the data dependent mode, cycle time 3 s between master scans, operating in positive ion mode. Fragmentation was induced by higher energy collisional dissociation (HCD) with collision energy set at 35 eV. MS raw data files were converted to mzML format (centroid mode) using the MSconvert tool of the software ProteoWizard (ver. 3.0.1957) [23]. Afterwards, mzML files were then analyzed using an OpenMS ver. 3.0 nodes operating within the open-source software platform KNIME^®^ (ver. 4.6) [24]. Peptides identification was done using a peptides identification approach combining the search engines as previously described [25]. The spectral library required by the SpectraST search engine was downloaded from the website www.peptideatlas.org (file NIST_mouse_IT_2012-04-21_7AA.splib). Peptide sequences were indexed through the OpenMS PeptideIndexer node, setting leucine/isoleucine equivalence. Protein inference was then carried out using the Protein Inference Analysis (PIA) algorithm using the default parameters set by the developers. Protein abundance estimates were calculated with prior generation of spectral feature by the node FeatureFinderMultiplex followed by PIA-assisted FDR-multiple scores estimation and filtering (combined FDR score < 0.01), their ID mapping and combination with peptide IDs, their subsequent alignment, grouping and normalization (e.g., MapAlignerIdentification, FeatureUnlabeledQT and ConsensusmapNormalizer nodes). Proteins and peptides label free quantification (LFQ) was then computed with the OpenMS ProteinQuantifier node based on intensities of the *n* = 3 most abundant identified peptides. The corresponding output files were read as tables of the CSVreader node output and exported into Microsoft Office Excel for further formatting and statistical elaboration. The abundances were Log_2_ transformed, normalized as previously described [26] and the proteins with at least three replicates were taken into consideration for statistical analysis. For proteome downstream analysis, Gene Ontology (GO) using the Database for Annotation, Visualization, and Integrated Discovery platform (DAVID, NIAID, Bethesda, MD) [27] and Ingenuity Pathway Analysis (IPA^®^, QIAGEN, Redwood City, CA) [28] were used.

### Histology

Aorta and liver were fixed in 4% paraformaldehyde (Sigma-Aldrich, Cat#252549) overnight at 4 °C, dehydrated at room temperature and paraffin-embedded. Tissue Sect. (5 μm) were deparaffinized and stained for hematoxylin and eosin (H&E) and Masson’s Trichrome (Bio-Optica, Cat#04-010802). The sections were acquired using the Axiovision Zeiss software, 5x magnification was used for aorta, and 10x magnification for the liver. For the aorta immunofluorescence staining was performed by overnight incubation at 4 °C as previously described [29], using primary antibody anti-α-SMA (Bio-Techne, Cat#NBP-67440, 1:200) or anti-Mac2 (Cedaelane, Cat#CL8942AP, 1:500). Thereafter, the sections were washed three times with PBS and incubated for 1 h with 2 mg/mL of Goat anti-Rabbit for α-SMA, anti-Rat for Mac2 for IgG (H + L) Cross-Adsorbed Secondary Antibody (Alexa Fluor 555) diluted 1:1000 in blocking solution, at RT, in agitation. Nuclei were stained through incubation with Hoechst diluted 1:4000 and coverslips sealed up over microscope slides with 2 µL of Fluoroshield ^TM^ mounting medium. Samples were visualised and acquired at the Zeiss confocal microscopy by LSM software (Zeiss ZEN), and images for the aorta were taken at 5x magnification and for the quantification ImageJ software was used.

### Statistical analysis

Data are expressed as the mean per group ± SEM and analyzed in accordance with the figure legends. For this study *p < 0.05, **p < 0.01 and ***p < 0.001 were considered significant. Comparisons within groups were made by using T-test for unpaired samples. For gene ontology (GO) terms, FDR < 0.05 was taken into consideration.

## Results

### ASGR1 deficiency reduces plasma lipids and atherosclerosis in experimental mice

**Fig. 1:**
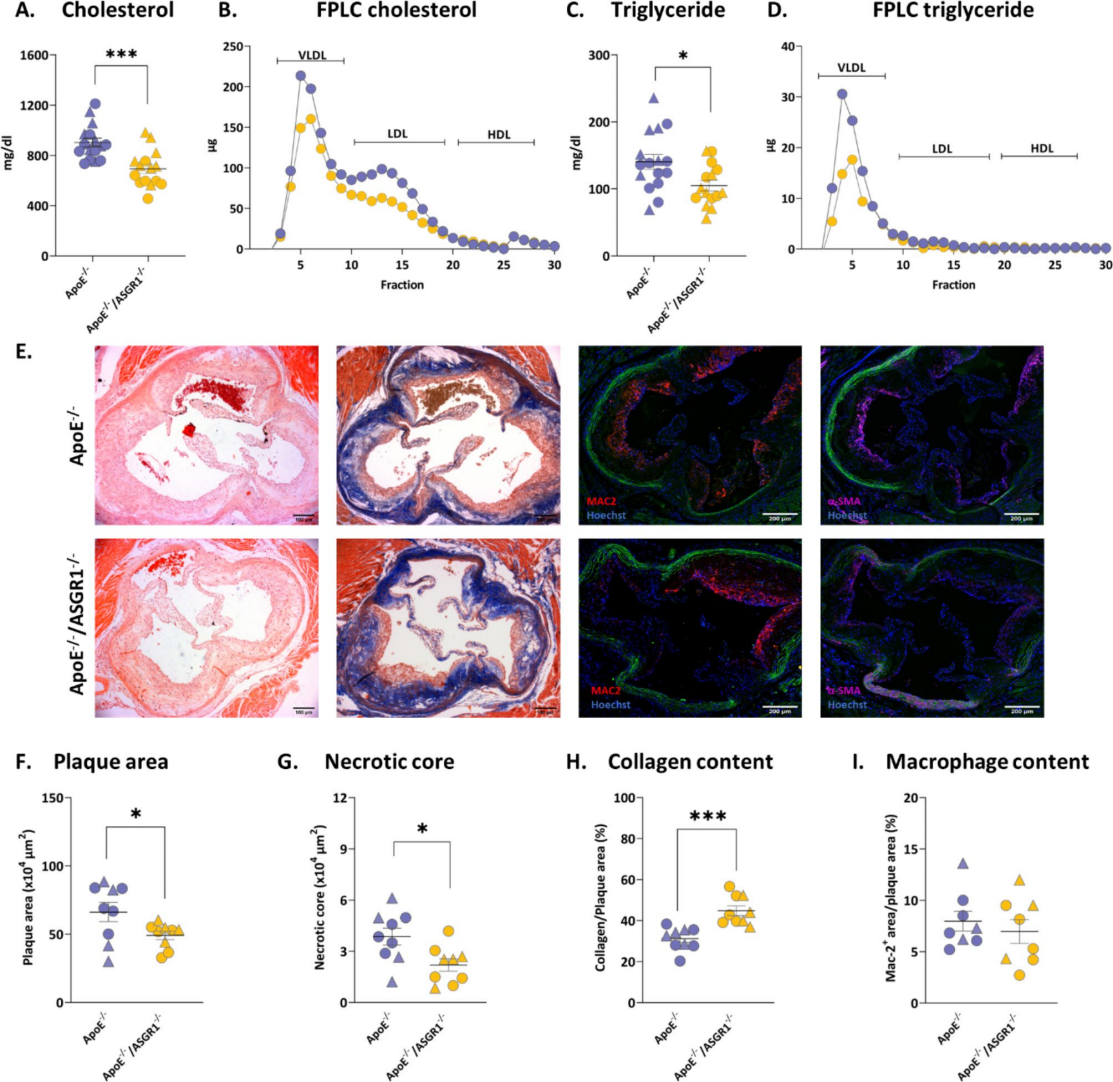
ASGR1 deficiency reduces plasma lipids and atherosclerosis in experimental mice. Plasma and aorta were collected after 16 weeks of western type diet (WTD) in male (represented by triangles) and female (represented by circles), in ApoE^−/−^ and ApoE^−/−^/ASGR1^−/−^ mice. The lipid levels were measure in fasting condition (**A**) The circulating cholesterol levels in ApoE^−/−^ (*n* = 16) and ApoE^−/−^/ASGR1^−/−^ (*n* = 15) mice (**B**) Cholesterol content measured in fractionated plasma in ApoE^−/−^ (*n* = 4) and ApoE^−/−^/ASGR1^−/−^ (*n* = 4) mice (**C**) The circulating triglyceride levels in ApoE^−/−^ (*n* = 16) and ApoE^−/−^/ASGR1^−/−^ (*n* = 15) mice (**D**) Triglyceride content measured in fractionated plasma in ApoE^−/−^ (*n* = 4) and ApoE^−/−^/ASGR1^−/−^ (*n* = 4) mice (**E**) Representative images at the aortic sinus stained for hematoxylin and eosin, Masson’s trichrome and immunofluorescence images for macrophage content (Mac2^+^, red) and smooth muscle cell (α-SMA^+^, magenta). Magnification 5x, scale bars 100–200 μm (**F**) Atherosclerotic plaque area (expressed in µm [2]) in ApoE^−/−^ (*n* = 9) and ApoE^−/−^/ASGR1^−/−^ (*n* = 9) mice (**G**) Necrotic core area (expressed in µm [2]) in ApoE^−/−^ (*n* = 9) and ApoE^−/−^/ASGR1^−/−^ (*n* = 9) mice (**H**) Collagen content (Masson’s trichrome staining expressed as % of positive stained area -blue staining- compared to total plaque area) in ApoE^−/−^ (*n* = 9) and ApoE^−/−^/ASGR1^−/−^ (*n* = 9) mice (**I**) Macrophage content (Mac2^+^ expressed as % of positive stained area -red staining- compared to total plaque area) in ApoE^−/−^ (*n* = 8) and ApoE^−/−^/ASGR1^−/−^ (*n* = 8) mice at the aortic sinus. The error bars show the mean ± SEM of male (represented by triangles) and female (represented by circle), ApoE^−/−^ and ApoE^−/−^/ASGR1^−/−^ mice. Each symbol in the graph represents an individual value. P values were calculated unpaired two-tailed Student’s t-test. *P < 0.05, **<0.01 and ***<0.001

To investigate the role of ASGR1 deficiency on plasma lipid profile during hypercholesterolemia, we fed ApoE^−/−^ and ApoE^−/−^/ ASGR1^−/−^ mice with western-type diet (WTD) for 16 weeks. ApoE^−/−^/ASGR1^−/−^ mice showed a 22% reduction in plasma cholesterol levels compared to ApoE^−/−^ (Fig. 1A, ApoE^−/−^ 892 ± 131 mg/dl and ApoE^−/−^/ASGR1^−/−^ 699 ± 141 mg/dl, *p* < 0.001). Further analysis using FPLC showed that cholesterol content was mainly reduced in VLDL and LDL fractions from ApoE^−/−^/ASGR1^−/−^ mice compared to that of ApoE^−/−^ mice (Fig. 1B). Also, plasma triglyceride levels were reduced in ApoE^−/−^/ASGR1^−/−^ mice compared to ApoE^−/−^ mice (Fig. 1C, ApoE^−/−^ 138 ± 44 mg/dl and ApoE^−/−^/ASGR1^−/−^ 109 ± 29 mg/dl), and mainly in the VLDL fraction (Fig. 1D). Next, we assessed whether these differences might have affected the atherosclerotic plaque development at the aortic sinus (Fig. 1E). ApoE^−/−^/ASGR1^−/−^ mice showed a significant reduction in the atherosclerotic plaque area (Fig. 1F, ApoE^−/−^ 662348 ± 210577 μm [2] and ApoE^−/−^/ASGR1^−/−^ 491372 ± 92229 μm [2]). Furthermore, ApoE^−/−^/ASGR1^−/−^ mice presented a reduction of the necrotic core area compared to ApoE^−/−^ mice (Fig. 1G, ApoE^−/−^ 38586 ± 14815 μm [2] and ApoE^−/−^/ASGR1^−/−^ 21929 ± 10885 μm [2]). Collagen content evaluation following Masson Trichrome staining showed that ApoE^−/−^/ASGR1^−/−^ mice present a significant increase of 44% in the plaque collagen content compared to ApoE^−/−^ mice (Fig. 1H, ApoE^−/−^ 31 ± 6% and ApoE^−/−^/ASGR1^−/−^ 45 ± 7%) indicating that ASGR1 deficiency might also promote a more stable plaque. Macrophage (Fig. 1I) and smooth muscle cells (Supp. Fig. S1A) content within the plaque were similar between the two animal models.

### ASGR1 deficiency increases circulating lymphoid cells in atherosclerotic mice

**Fig. 2:**
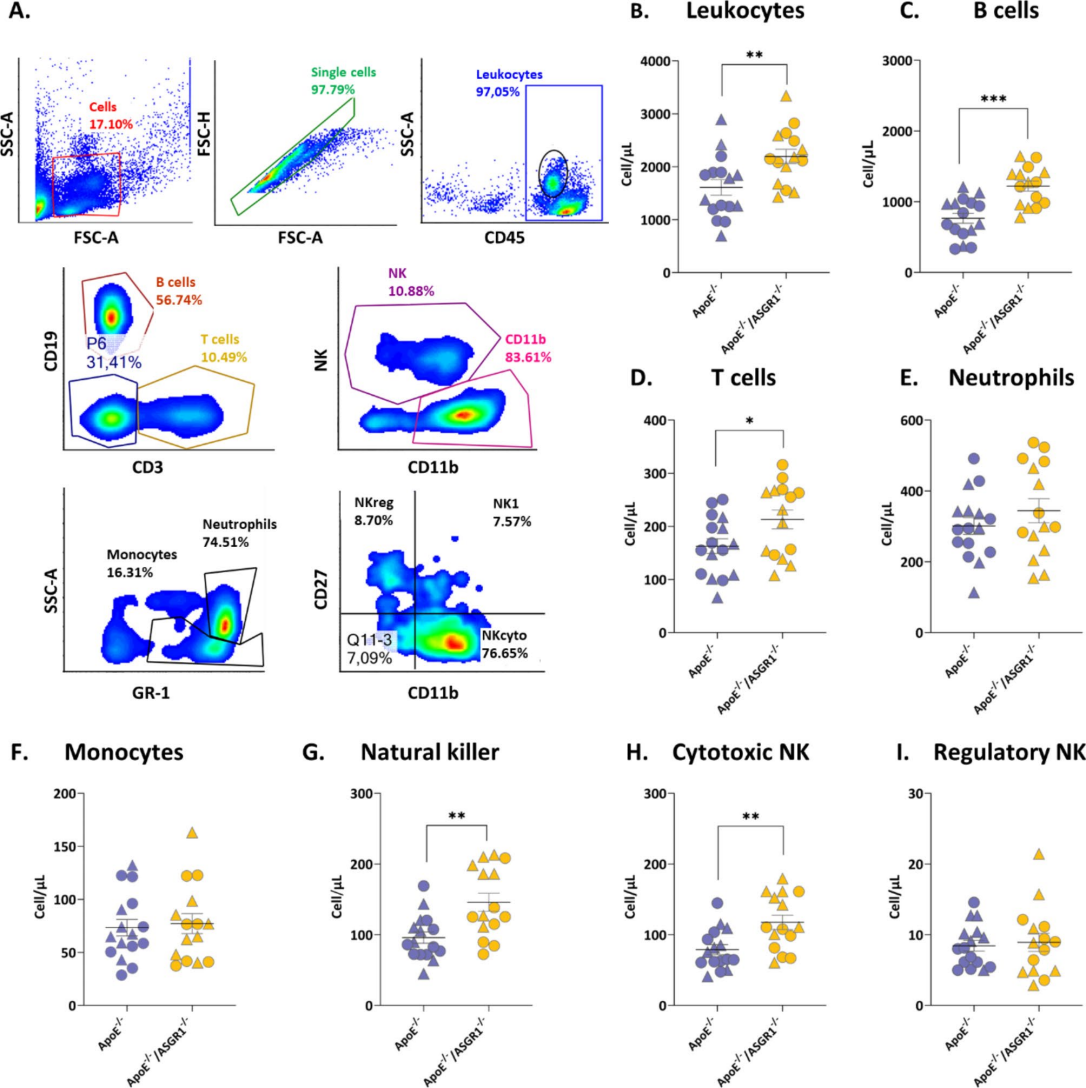
ASGR1 deficiency increases circulating immune cell surveillance in atherosclerotic mice. (**A**) Flow cytometry gating strategy for blood immunophenotyping: cells were gated based on dimensions (SSC-H vs. FSC-H) and doublets were excluded by the analysis (SSC-A vs. SSC-H). Immune cells, in ApoE^−/−^ (*n* = 16) and ApoE^−/−^/ASGR1^−/−^ (*n* = 15) mice, male (represented by triangles) and female (represented by circles), were identified as following: Leukocytes were gated on CD45^+^ positive cells (**B**), B cells (CD19^+^, CD3^−^) (**C**), T lymphocytes (CD19^−^, CD3^+^) (**D**), monocytes and neutrophils (NK1^−^, CD11b^+^, GR-1^+^) (**E-F**), Natural killer cells (NK, CD19^−^, CD3^−^,CD11b^−^,NK1^+^) (**G**), Cytotoxic NK cells (CD27^+^,CD11b^−^ among NK1^+^) (**H**), Regulatory NK cells (CD27^+^, CD11b^−^ among NK1^+^) (**I**). Data are expressed as absolute count. The error bars show the mean of cell/µL ± SEM of male (triangles) and female (circles), ApoE^−/−^ and ApoE^−/−^/ASGR1^−/−^ mice. Each symbol in the graph represents an individual value. P values were calculated unpaired two-tailed Student’s t-test. *P < 0.05, **<0.01 and ***<0.001

We next investigated whether ASGR1 deficiency might also impact the immune profile (Fig. 2A). Surprisingly, ApoE^−/−^/ASGR1^−/−^ mice presented a significant increase in circulating leukocytes levels compared to ApoE^−/−^ mice (Fig. 2B). Consequently, this was followed also by a significant increase in the levels of B and T lymphocytes in circulation (Fig. 2C and D respectively). While the number of neutrophils and monocytes was similar in the two animal models (Fig. 2E and F respectively) as was the number of dendritic cells (DC, Supp. Fig. S1B).

Interestingly also the number of natural killer (NK) cells (Fig. 2G) and more specifically that of cytotoxic natural killer cells (Fig. 2H), but not that of regulatory NK (Fig. 2I) or of NK1 (Supp. Fig. S1C), was significantly increased in ApoE^−/−^/ASGR1^−/−^ compared to ApoE^−/−^ mice. These findings support the hypothesis that ASGR1 deficiency reduces atherosclerosis by decreasing plasma lipids levels independently of an effect on innate immune cells while the counts of adaptive immune cells appear to be increased under ASGR1 deficient conditions.

### ASGR1 deficiency impacts plasma O-acetylation of sialic acid in atherosclerotic mice

**Fig. 3:**
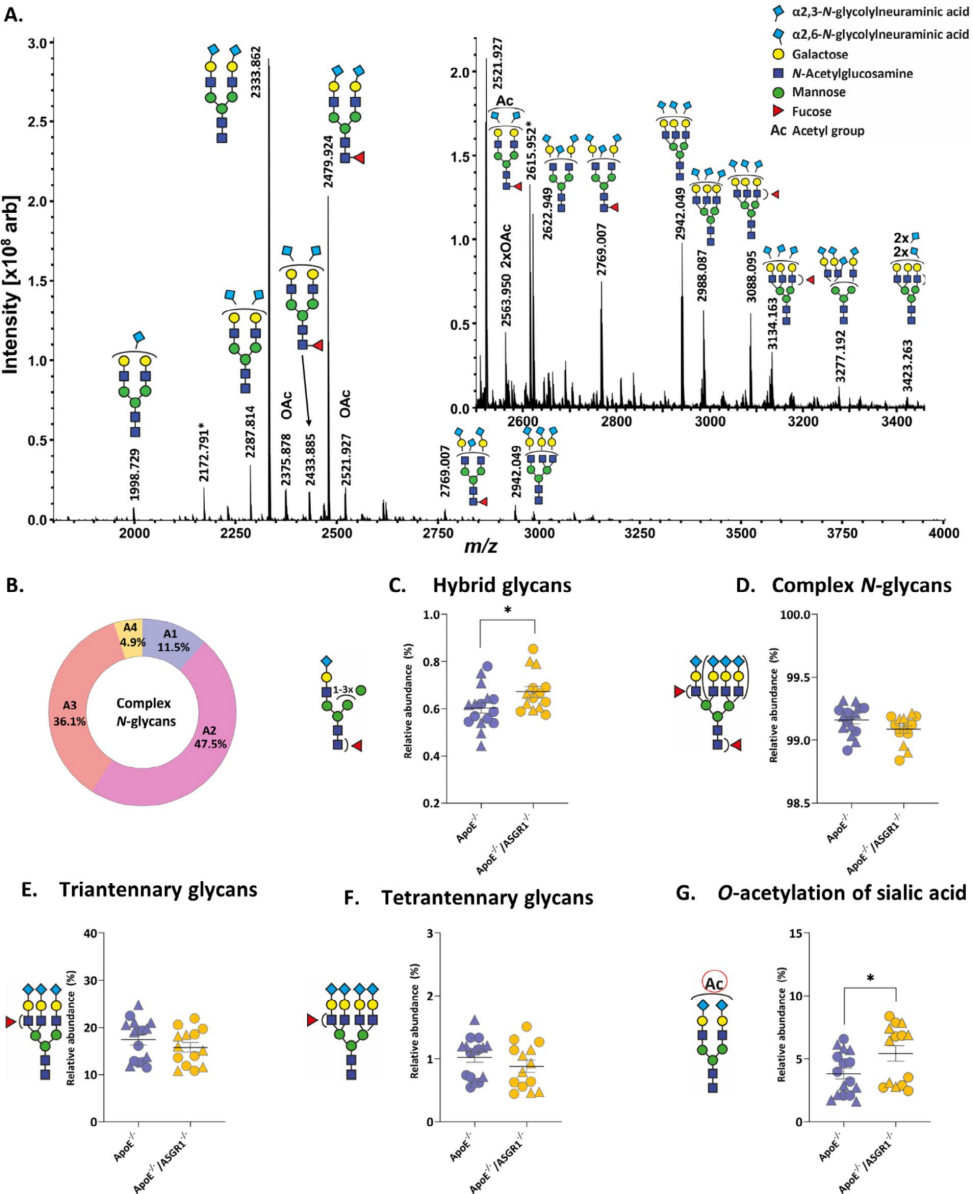
ASGR1 deficiency impacts plasma O-acetylation of sialic acid in atherosclerotic mice. Plasma *N*-glycome in male (represented by triangles) and female (represented by circles), ApoE^−/−^ (*n* = 16) and ApoE^−/−^/ASGR1^−/−^ (*n* = 14) mice after 16 weeks of western type diet (WTD) (**A**) MALDI-FT-ICR-MS spectrum depicting annotated *N*-glycans in ApoE^−/−^/ASGR1^−/−^ mice. *N*-Glycans are detected as [M + Na]^+^ ions and the descriptions of the glycan cartoons are shown in the right upper panel (**B**) Percentage of complex *N*-glycans based on the antennary (**C**) Hybrid glycans (**D**) Complex *N*-glycans (**E**) Tri-antennary glycans (**F**) Tetra-antennary glycans (**G**) O-acetylation of sialic acid. The error bars show the mean of relative abundance (%) ± SEM of male (triangles) and female (circles), ApoE^−/−^ and ApoE^−/−^/ASGR1^−/−^ mice. Each symbol in the graph represents an individual value. P values were calculated unpaired two-tailed Student’s t-test. *P < 0.05, **<0.01 and ***<0.001

The observation above raises the possibility that changes in plasma *N*-glycome due to ASGR1 deficiency might promote different inflammatory response independently of the lipid lowering effects in an atheroprone setting. As the main function of ASGR1 is the clearance of desialylated glycoproteins in circulation, we first profiled the plasma *N*-glycome to evaluate whether the absence in ASGR1 might influence the *N*-glycan turnover (Fig. 3A). A total of 72 *N*-glycan compositions were identified in plasma of ApoE^−/−^ and ApoE^−/−^/ASGR1^−/−^ mice (Supp. Tab. S2).

By integrating common structural glycan features we evaluated the results according to fifteen glycosylation traits (Supp. Tab. S3). Based on these traits, the most prevalent structures were complex glycans (> 99%), of which 47.5% were di-antennary glycans followed by 36.1% tri-antennary glycans and then by mono-/tetra-antennary glycans as shown in Fig. 3B. As the ligand binding capacity of ASGR1 to remove the glycoproteins increases significantly and the Gal moieties increases from mono- to tri-antennary structures, we suspected to have an accumulation of tri-antennary glycans in ApoE^−/−^/ASGR1^−/−^ mice. Interestingly, we did not observe changes in either mono-/diantennary (Supp. Fig. S2D- S2E) and tri-/tetra-antennary glycans regarding sialic acid abundance or linkage in both genotypes (Supp. Fig. S2F and S2G). As expected, 98% of the complex *N*-glycans presented in both genotypes exhibited complete galactosylation. This suggests that desialylated glycoproteins with exposed Gal are removed from circulation before further trimming by galactosidases can occur. In parallel, we performed a linkage-specific sialic acid derivatization analysis to detect α2,6-linked sialylation and α2,3-linked sialylation. Given the minimal changes observed in *N*-glycome between ApoE^−/−^/ASGR1^−/−^ and ApoE^−/−^, we evaluated the presence of O-acetylation (O-Ac) as a sialic acid substituent, considering that it can protect glycoproteins from degradation and clearance. Interestingly, ApoE^−/−^/ASGR1^−/−^ mice presented an increase in O-Ac compared to the ApoE^−/−^ mice (Fig. 3G), suggesting a possible implication of acetyl group donor, acetyl-coenzyme A (Acetyl-CoA).

### ASGR1 deficiency affects cholesterol content in the liver of atherosclerotic mice

**Fig. 4:**
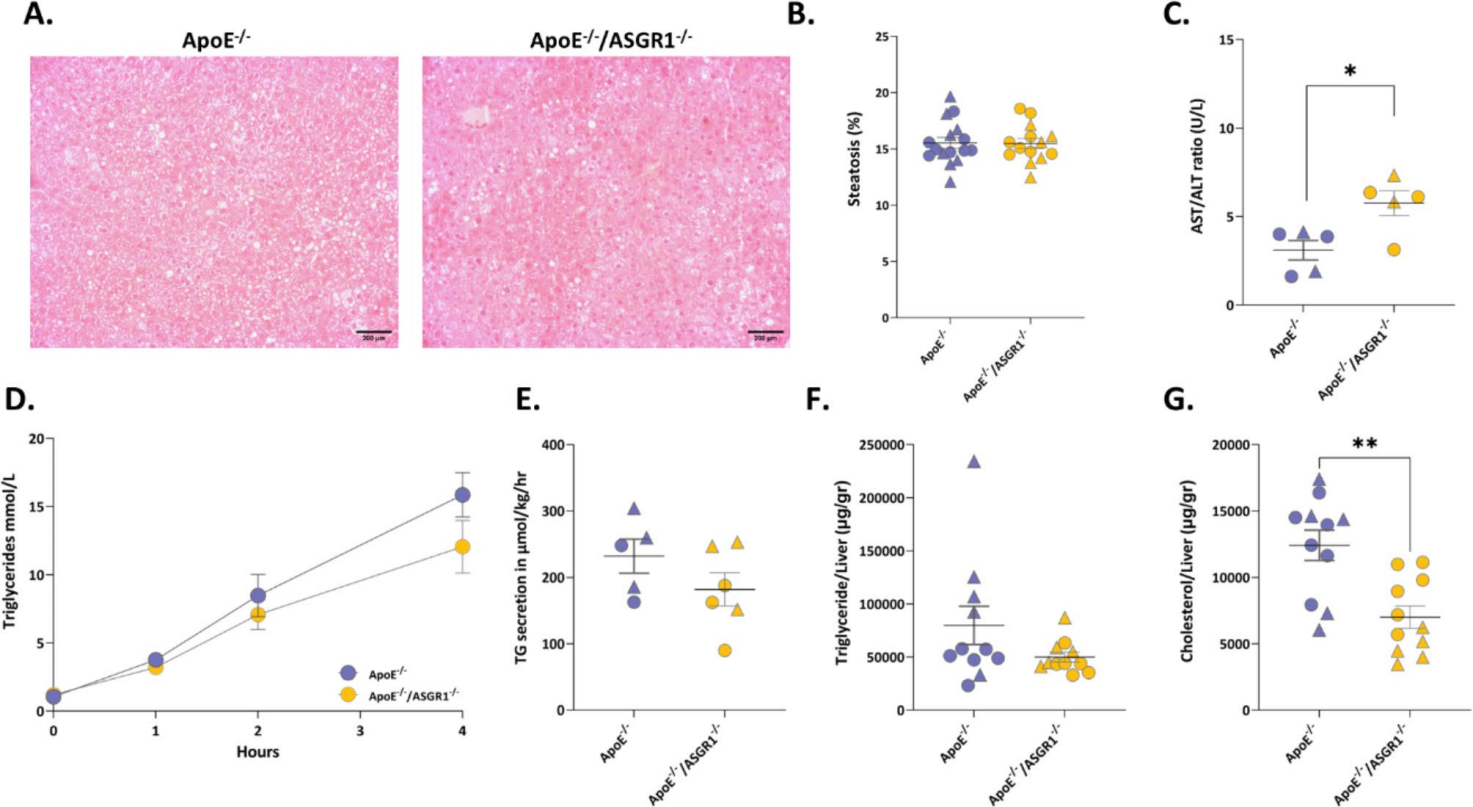
ASGR1 deficiency affects cholesterol content in the liver of atherosclerotic mice. Liver and plasma were collected after 16 weeks of western type diet (WTD) in male (represented by triangles) and female (represented by circles), ApoE^−/−^ and ApoE^−/−^/ASGR1^−/−^ mice. (**A**) Representative images of liver sections stained with H&E. Scale bar, 200 μm (**B**) Liver lipid droplet quantification in ApoE^−/−^ (*n* = 16) and ApoE^−/−^/ASGR1^−/−^ mice (*n* = 14) (**C**) Plasma ALT/AST ratio in ApoE^−/−^ (*n* = 5) and ApoE^−/−^/ASGR1^−/−^ mice (*n* = 5) (**D**) Circulating plasma triglyceride levels (mmol/L) in mice following poloxamer injection, in ApoE^−/−^/ASGR1^−/−^ mice (*n* = 6) compare to ApoE^−/−^ (*n* = 5) (**E**) Triglyceride (TG) secretion rate in µmol/kg/hr, calculated from (*D*). (**F**) Liver triglyceride levels in ApoE^−/−^ (*n* = 11) and ApoE^−/−^/ASGR1^−/−^ mice (*n* = 11) (**G**) Liver cholesterol levels in ApoE^−/−^ (*n* = 11) and ApoE^−/−^/ASGR1^−/−^ mice (*n* = 11). Each symbol in the graph represents an individual value of male (triangles) and female (circles), ApoE^−/−^ and ApoE^−/−^/ASGR1^−/−^ mice. P values were calculated using the unpaired two-tailed Student’s t-test. *P < 0.05, **<0.01 and ***<0.001

Decreased plasma lipid levels coupled with changes in plasma O-Ac of sialic acid prompted us to investigate whether this profile could reflect increased liver steatosis/inflammation in ASGR1 deficiency. We therefore investigated liver morphology (Fig. 4A) and observed a similar lipid accumulation and morphology (Fig. 4B, Supp. Fig. S3A) together with a similar liver weight between genotypes (Supp. Fig. S3B). Nevertheless, the AST/ALT ratio in the plasma was significantly increased in ApoE^−/−^/ASGR1^−/−^ mice compared to ApoE^−/−^ mice (Fig. 4C). Also the expression of liver injury markers such as FABP1, CYP2E1, CES1D, LGAL3 (Supp. Fig. S3C) was increased in ApoE^−/−^/ASGR1^−/−^ mice. Coupled together, these findings support the hypothesis that decreased plasma cholesterol levels could be the result of decreased lipoprotein production in the liver of ApoE^−/−^/ASGR1^−/−^ mice. To test this possibility, we treated the experimental groups with poloxamer to inhibit the lipolysis of triglyceride-rich lipoproteins, thus allowing to monitor lipoprotein production (Fig. 4D). We observed a trend toward reduced plasma triglyceride levels following poloxamer injection in ApoE^−/−^/ASGR1^−/−^ compared to ApoE^−/−^ mice, however, these changes were not statistically significant (Fig. 4E). Next, we evaluated the lipid content in the liver of ApoE^−/−^/ASGR1^−/−^ mice compared to ApoE^−/−^ mice and observed that triglyceride levels were similar between the genotypes (Fig. 4F) while the cholesterol levels were significantly reduced in ApoE^−/−^/ASGR1^−/−^ mice compared to ApoE^−/−^ mice (Fig. 4G) suggesting that ASGR1 under WTD specifically reduces the cholesterol content in the liver.

### ASGR1 deficiency enhances lipid catabolism in atherosclerotic mice

**Fig. 5:**
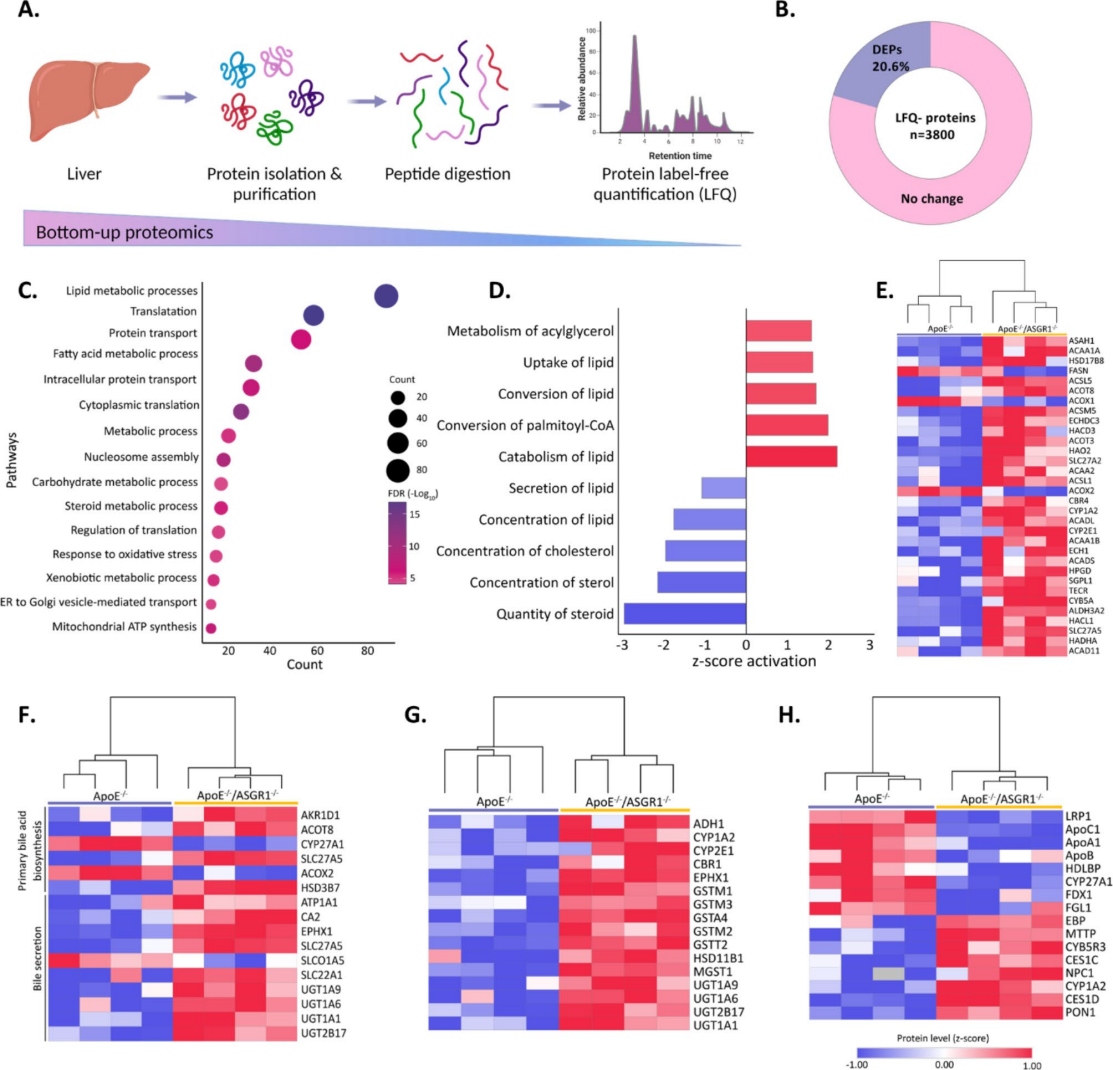
ASGR1 deficiency enhances lipid catabolism in atherosclerotic mice. Liver was collected after 16 weeks of western type diet (WTD) in ApoE^−/−^ (*n* = 4) and ApoE^−/−^/ASGR1^−/−^ mice (*n* = 4) (**A**) Scheme depicting proteomic strategy used to identify and label-free quantify (LFQ) liver proteins in ApoE^−/−^ and ApoE^−/−^/ASGR1^−/−^ mice. Created with BioRender. (**B**) Untargeted liver proteome showing differentially expressed proteins (p-value < 0.05) (**C**) Top 15 pathways based on the gene ontology term for biological processes on the differentially expressed proteins (DEPs) in the liver of ApoE^−/−^/ASGR1^−/−^ mice compare to ApoE^−/−^ (**D**) Lipid metabolic processes, based on IPA pathways, of differently expressed proteins, the inhibited pathways are highlighted in blue (z-score < -2) and activated pathways are highlighted in red (z-score > 2) (**E-H**) Heatmap with hierarchical clustering representing the protein level (z-score transformed) for the fatty acid metabolic processes (**E**), Primary bile acid biosynthesis and bile secretion (**F**), Xenobiotics by CYP P450 (**G**), and Cholesterol metabolism (**H**) in the liver of ApoE^−/−^/ASGR1^−/−^ mice compare to ApoE^−/−^. P values were calculated unpaired two-tailed Student’s t-test. *P < 0.05, **<0.01 and ***<0.001

To further explore this possibility, we profiled the liver proteomic signature of mice, after 16 weeks of WTD (Fig. 5A). This analysis identified a distinct liver proteomics pattern in ApoE^−/−^/ASGR1^−/−^ compared to ApoE^−/−^/ mice (Supp. Fig S3D, Supp. Tab. S4). Among the 3800 proteins that were identified, 20.6% were differentially expressed between the two groups (DEPs, *p* < 0.05, Fig. 5B). DEPs showed significant overlap in gene ontology (GO) terms for lipid metabolic processes including cholesterol and fatty acid metabolism, protein transport, carbohydrate metabolic processes and xenobiotic metabolic processes (depicted the top 15 pathways in Fig. 5C, Supp. Tab S5), suggesting that ASGR1 deficiency exerts a broader molecular impact, which extends beyond lipid metabolism.

Next, we conducted a detailed analysis of lipid metabolic processes using IPA Qiagen software. We observed significant alterations in pathways affecting lipids and cholesterol levels (Fig. 5D, Supp. Tab. S6). Conversely there was a significant increase in the metabolism of acylglycerol, lipid uptake as well as lipid catabolism, and conversion to palmitoyl-CoA suggesting that triglycerides in the liver are likely being utilized to produce energy. Furthermore, as shown in the heatmap (Fig. 5E), the liver of ApoE^−/−^/ASGR1^−/−^ presented a significant upregulation of all proteins associated with fatty acid metabolic pathways, AMPK signaling, and carbohydrate metabolic processes (Supp. Fig. S3E-G), including galactose galactokinase (GALK1), the major enzymes that catabolize galactose and galactose mutarotase (GALM), which maintains the balance between α and β anomers of galactose, lysosomal β-hexosaminidase B (HEXB) and lysosomal β-mannosidase, suggesting that glycans do enter in the liver of ApoE^−/−^/ASGR1^−/−^ for further lysosomal enzymatic processing.

Within the pathways that appeared to be significantly upregulated (FDR < 0.05), we observed those related to the metabolism of xenobiotics by CYP-P450, primary bile acid biosynthesis and bile secretion (Fig. 4F) were activated, with several members of cytochrome P450 complex (CYP1A2, CYP2E1), UDP-glucuronyltransferases (UGT1A9, UGT1A6, UGT2B17, UGT1A1), glutathione S-transferases (GSTM1, GSTM3, GSTA4, GSTN2, GSTT2, MGST1), epoxide hydrolases (EPHX1) and solute carrier (SLC) transporters such as SLC22A1 being significantly upregulated (Fig. 4G). Interestingly, while lipid catabolism is upregulated, cholesterol metabolism related proteins such as low-density lipoprotein receptor-related protein 1 (LRP1), high density lipoprotein binding protein (HDLBP), and apolipoproteins C1, A1, B as well as CYP27A1, which catalyzes the degradation of cholesterol to bile acids, were downregulated (Fig. 4H). In summary, our data establish ASGR1 as a key determinant of modulating lipid and lipoprotein metabolism while also influencing protein and glycan signaling pathways.

## Discussion

The development and progression of atherosclerotic lesions are triggered by the retention of lipoproteins which in turn activate a low-grade inflammation characterized by infiltration and a gradual accumulation of inflammatory cells such as macrophages [30]. Different studies in human and mouse models have shown that ASGR1 deficiency leads to a notable decrease in lipid levels which in turn corresponds to a reduction in CVD risk [5, 12, 14]. We have further extended these findings showing that under hypercholesterolemic conditions, ASGR1 deficiency results in lower plasma lipid levels with a reduction in the atherosclerotic lesions coupled to a smaller necrotic core and increased collagen content. Furthermore, given the role of ASGR1 in modulating the recognition of desialylated glycoproteins, which are highly abundant during infection and inflammatory conditions [15], we focused our attention on the investigation of the effect of ASGR1 deficiency on the immunoinflammatory response observed during atherogenesis and appreciated an increase in the inflammatory signature while the plasma *N*-glycome remained stable during atherogenesis. In contrast, CYP-P450 proteins involved in lipids and xenobiotics metabolism and bile acid synthesis were increased in the liver of those mice. 

We further showed that ApoE^−/−^/ASGR1^−/−^ mice present a 22% reduction in circulating lipid levels even after 16 weeks of WTD compared to ApoE^−/−^. This reduction resulted in a lower atherosclerotic plaque development and reduced necrotic core size at the aortic sinus. Additionally, the atherosclerotic plaques of ApoE^−/−^/ASGR1^−/−^ mice presented a significant increase of 44% in collagen content compared to ApoE^−/−^ mice, indicating that ASGR1 deficiency results in improved plaque structural integrity. Interestingly, macrophages which account for the majority of leukocytes in atheroma, were not different in ApoE^−/−^/ASGR1^−/−^ mice.

Nevertheless, ApoE^−/−^/ASGR1^−/−^ mice displayed increased levels of lymphoid cells including B cells and T cells in blood, along with a pronounced increase in natural killer (NK) cells particularly in the cytotoxic NK subset suggesting an activation of circulating immune cells. The absence of significant changes in macrophages and smooth muscle cells in the aorta suggests that these systemic immune changes may not directly impact the local cellular environment within the plaque. These findings warrant further investigation to understand the activation status of these immune cells and clarify whether this could impact liver immune response.

In addition to the immunophenotype in ApoE^−/−^/ASGR1^−/−^ mice, we profiled circulating *N*-glycome. Given the ASGR1 affinity for complex *N-*glycans, which can increase up to 1000-fold as galactose (Gal) moieties increase from mono- to tri-antennary, we expected a corresponding increase in antennarity of *N*-glycans [6]. Additionally, it is known that α2,3-sialylation of terminal Gal prevents the recognition and clearance of circulating glycoproteins by ASGR1 [31], thus we expected an accumulation of α2,6-sialylation in the absence of the receptor. Also, in the context of atherosclerosis, it was shown that the increase in α2,3-sialylation in ApoE^−/−^ mice potentially increases the size of atherosclerotic areas and the numbers of macrophages in the lesion, without affecting plasma cholesterol levels [32]. However, we did not observe any differences between genotypes in overall *N*-glycome, including glycoproteins with α2,6-sialylation or exposed Gal which are cleared from circulation even in the absence of ASGR1. The stable *N*-glycome in circulation in ApoE^−/−^/ASGR1^−/−^ mice suggests that the liver maintains the glycoprotein turnover. This stability may be due to the presence of functionally redundant receptors with overlapping specificity to ASGR1 [33, 34]. However, another route of glycans, to enter the liver is via the hepatic portal vein as the hepatocytes can uptake freely from the circulation, if needed as a substrate for *N*-glycan synthesis [35]. Interestingly, in the liver of ApoE^−/−^/ASGR1^−/−^ mice, we observed a significant increase in the protein level of galactokinase (*GALK1*), the major enzyme that catabolizes Gal through the Leloir pathway to form glucose-1-phosphate, which can enter glycolysis or gluconeogenesis, and galactose mutarotase (*GALM*), which maintains the balance between α and β anomers of galactose, suggesting that ASGR1 cannot be a limiting step for Gal as a glycan moieties.

In contrast to the general absence of circulating *N*-glycome changes, we observed a relative accumulation of O-acetylation (O-Ac) of sialic acid in ApoE^−/−^/ASGR1^−/−^ mice which has been reported previously also in ASGR1 deficient mice, fed on high-fat diet [36]. Interestingly, O-Ac takes place in the Golgi apparatus and is influenced by the availability of the acetyl group donor, acetyl-coenzyme A (Acetyl-CoA), the key molecule which generates the energy in the liver [37–39]. Notably, the increased xenobiotics metabolism in the liver has a propensity to form conjugates with CoA. While the vast majority of these conjugates bound to carnitine and enter in β-oxidation for ATP production, another part is used to facilitate transport across hepatocellular membranes into bile or in circulation leading to protein acetylation [40]. Also, modification of sialic acid residues by O-Ac, can inhibit the action of sialidases, therefore protecting sialic acid from degradation [41]. Interestingly, pharmacological inhibition of sialidases 1 (NEU1) and sialidase 3 (NEU3) in ApoE^−/−^ mice has been found to reduce atherosclerotic lesion sizes in the aortic root [42]. This suggests that modifying sialidase activity, either through O-acetylation or pharmacological inhibition, could be a potential therapeutic strategy for reducing atherosclerosis. Whether the observed increase in O-Ac of sialic acid in ApoE^−/−^/ASGR1^−/−^ is due to the disbalanced glycoprotein biosynthesis or due to changes in glycan remodeling in plasma warrants additional investigation.

Interestingly, in humans, ASGR1 loss of function has been associated with elevated levels of alkaline phosphate (ALP) in circulation which have been attributed to the compromised clearance of desialylated glycoproteins [5]. However, our data suggest that even when *N*-glycome profile is not altered, the increased ALT/AST ratio in ApoE^−/−^/ASGR1^−/−^ mice could mark changes in liver function. Moreover, our group and others showed that ASGR1 deficiency promotes liver damage in mice and humans with obesity [43–45]. Interestingly, the susceptibility of ASGR1 deficient mice to liver injury is considered to be a consequence of “drug-induced liver injury” mediated via CCL4 ^44^. To further evaluate the functional impact of ASGR1 deficiency in the liver of ApoE^−/−^/ASGR1^−/−^ we performed untargeted liver proteomics. This analysis revealed a significant increase in lipid uptake and catabolism along with a significant increase in several cytochrome P450 proteins, including the bile excretion pathway suggesting an overall acceleration in the processing and excretion of lipids as well as liver amino-acid degradation. These findings support the Wang et al. hypothesis that ASGR1 deficiency upregulates LXRα which promotes cholesterol excretion and decreases lipid levels and reduces amino-acid levels in lysosomes, thereby inhibiting mTORC1 and activating AMPK [12]. Overall, we observed a reduction in ApoB-containing lipoproteins and cholesterol levels in the circulation of ApoE^−/−^/ASGR1^−/−^ mice, it is important to note that changes in xenobiotic receptors go beyond lipid metabolism, leading to complex alterations in fatty acid metabolism, carbohydrate metabolic and intracellular protein transport from endoplasmic reticulum to Golgi compartment. We should acknowledge, within the limitations of our study, that we have addressed atherosclerosis at a single time point (16 weeks of WTD diet) and the evaluation at a shorter time point might contribute to a better understanding the impact of ASGR1 deficiency on atherosclerosis and liver function.

Finally, while targeting hepatic ASGR1 would represent an intriguing lipid-lowering strategy for dyslipidemia thus reducing the burden of atherosclerotic cardiovascular diseases, the potential adverse effects including hepatic cardiometabolic and inflammation consequences, should be taken into consideration before starting the development of pharmacological strategies inhibiting ASGR1.

## Supplementary Information

Below is the link to the electronic supplementary material.

Supplementary Material 1

## Data Availability

No datasets were generated or analysed during the current study.

## Abbreviations

ALT: Alanine Aminotransferase
AST: Aspartate Aminotransferase
ASGPR: Asialoglycoprotein receptor
ASGR1: Asialoglycoprotein receptor 1
ASGR1^−/−^: Asialoglycoprotein deficient mice
ApoE^−/−^: Apolipoprotein E deficient mice
CVD: Cardiovascular disease
Chol: Cholesterol
CYP-P450: Cytochrome P450
DEPs: Differentially expressed proteins
Gal: Galactose
GO: Gene ontology
LDL: Low-density lipoprotein
LFQ: Label-free quantification
LXR: Liver X receptor
GalNAc: *N*-acetylgalactosamine
O-Ac: O-acetylation of sialic acid
SREBP: Sterol regulatory element-binding protein
TGs: Triglycerides
WTD: Western type diet
