# Supplementary material for: ASGR1 deficiency improves atherosclerosis but alters liver metabolism in ApoE-/- mice
Source: Cardiovasc Diabetol. 2024 Nov 30;23:428. doi: 10.1186/s12933-024-02507-5 (PMC11608471; doi:10.1186/s12933-024-02507-5)
Supplement: Supplementary file 1 — Supplementary Material 1 [file 12933_2024_2507_MOESM1_ESM.pdf]

Supplementary data

ASGR1 deficiency improves atherosclerosis but alters liver metabolism in ApoE$^{-/-}$ mice in ApoE$^{-/-}$ mice

Monika Svecla[1], Annalisa Moregola[1], Lorenzo Da Dalt[1],Jasmine Nour[1], Andrea Baragetti[1], Patrizia Uboldi[1], Alessandra Idini[1], Manfred Wuhrer[2], Giangiacomo Beretta[3], David Falck[2],, Fabrizia Bonacina[1], Giuseppe Danilo Norata [1,4]

[1] Department of Pharmacological and Biomolecular Sciences, Università degli Studi di Milano, Milan, Italy. [2] Center for Proteomics and Metabolomics, Leiden University Medical Center, Leiden, The Netherlands. [3]Department of Environmental Science and Policy, Università degli Studi di Milano, Milan, Italy.[4] Centro SISA per lo studio dell'Aterosclerosi, Ospedale Bassini, Cinisello Balsamo, Italy.

Corresponding author:

Giuseppe Danilo Norata, Professor of Pharmacology, University of Milan,Via Balzaretti 9, 20133 Milan, Italy. E-mail: Danilo.Norata@unimi.it

Supplement Figure S1

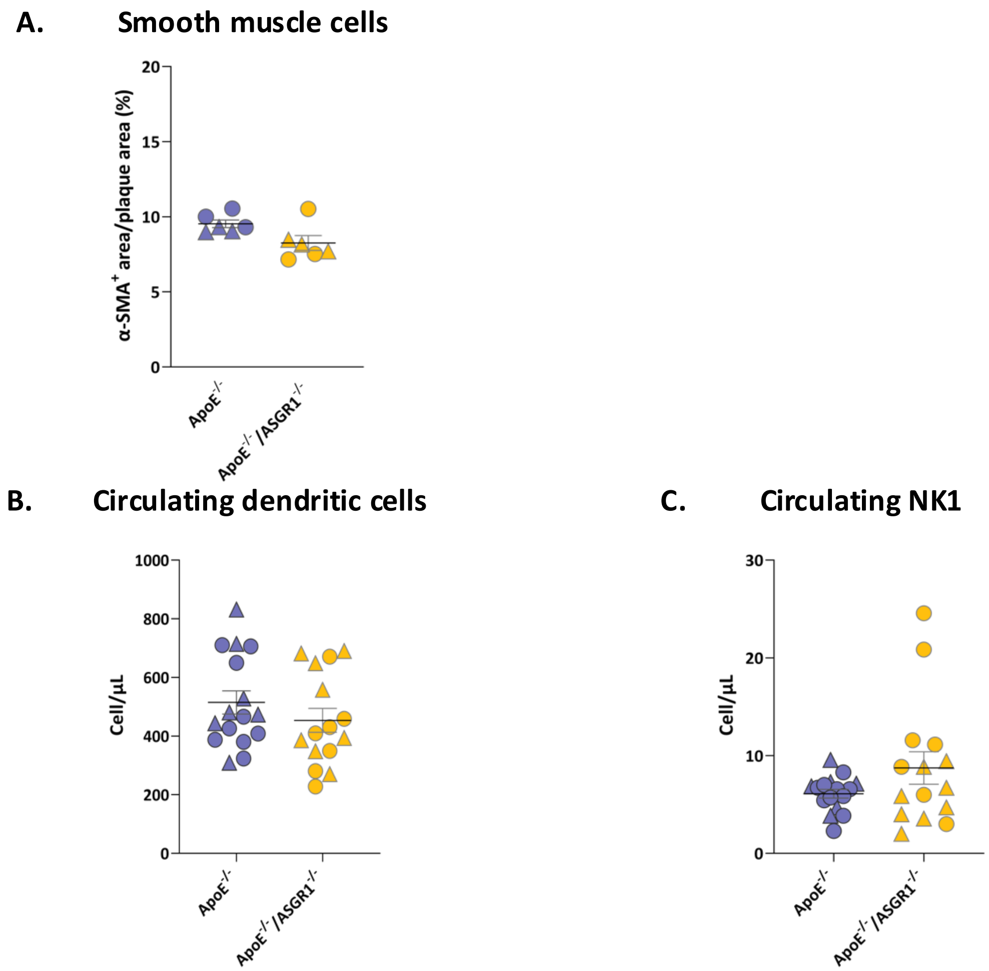

Supplementary Figure S1 – ASGR1 deficiency role on SMC in aortic arch and circulating immune cells in ApoE$^{-/-}$ mice. Aorta and blood for immunophenotyping were collected after 16 weeks of western diet (WTD) in male and female, ApoE$^{-/-}$ and ApoE$^{-/-}$/ASGR1$^{-/-}$ mice. (***A***) Smooth muscle cell content (α-SMA^{+} expressed as % of positive stained area compared to total plaque area) at the aortic sinus in ApoE$^{-/-}$ (n=6) and ApoE$^{-/-}$/ASGR1$^{-/-}$ (n=6) mice. (***B***) Circulating dendritic cells (DC, CD 11c^{+})(***C***) Circulating NK cells (CD27^{+},CD11b^{+} among NK1^{+}). (***B-C***) Data for the blood immunophenotyping are expressed as absolute count in ApoE$^{-/-}$ (n=16) and ApoE$^{-/-}$/ASGR1$^{-/-}$ (n=15) mice. The error bars show the mean of cell/µL ± SEM of male (represented by triangles) and female (represented by circle), ApoE$^{-/-}$ and ApoE$^{-/-}$/ASGR1$^{-/-}$ mice. P values were calculated unpaired two-tailed Student's t-test. *P < 0.05, **<0.01 and ***<0.001. Each symbol in the graph represents an individual value.

Supplement Figure S2

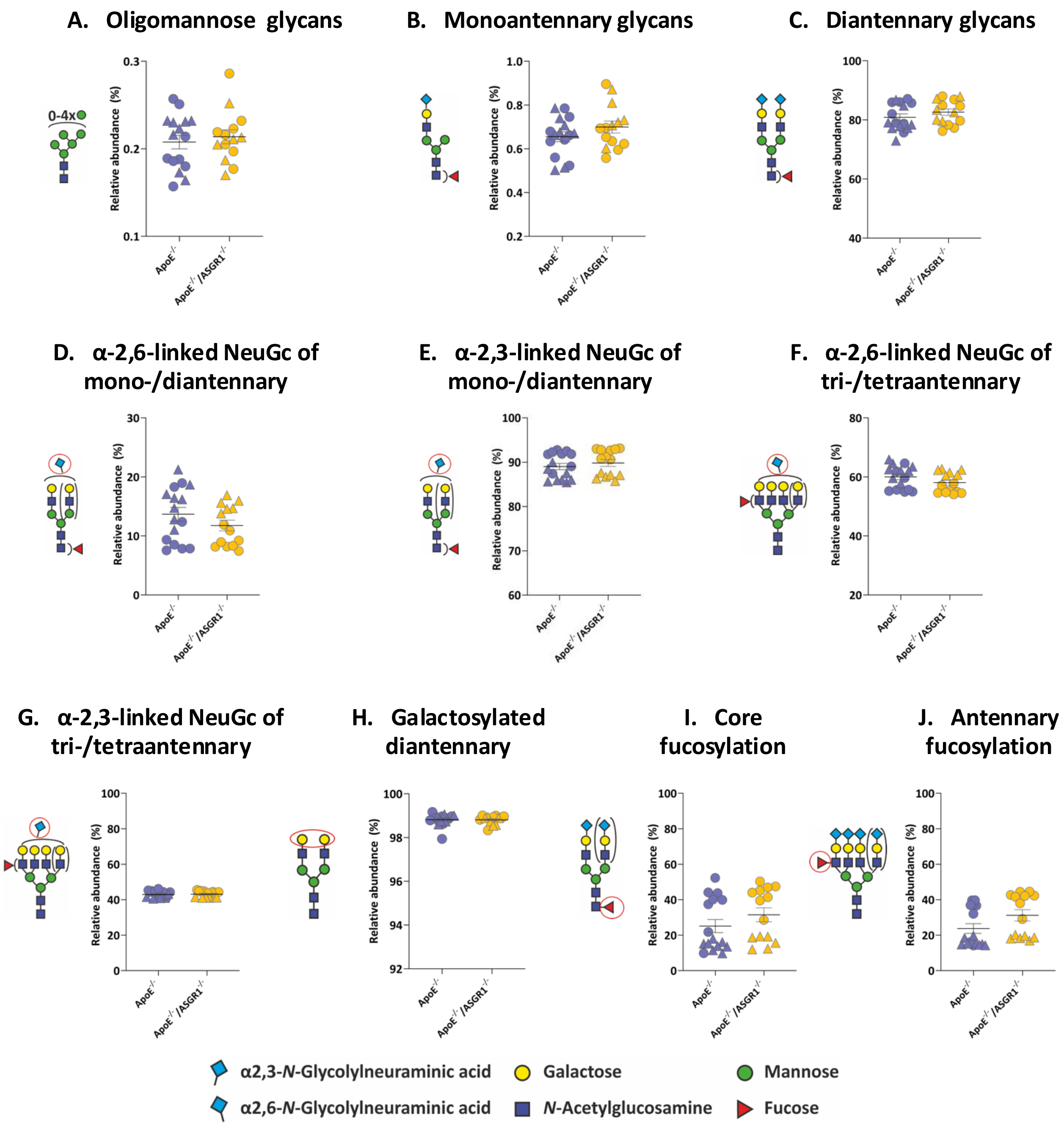

Supplementary Figure S2 – Glycosylation traits in ASGR1 deficency in atherosclerotic mice. Plasma was collected after 16 weeks of western diet (WTD) in male and female, ApoE$^{-/-}$ and ApoE$^{-/-}$/ASGR1$^{-/-}$ mice. (***A***) Oligomannose, (***B***) Monoantennary glycans, (***C***) Diantennary glycans, (***D***) α2,6-linked *N*-Glycolylneuraminic acid of mono-/diantennary glycans, (***E***) α2,3-linked *N*-Glycolylneuraminic acid of mono-/diantennary glycans, (***F***) α2,6-linked *N*-Glycolylneuraminic acid of tri-/tetraantennary glycans, (***G***) α2,3-linked *N*-Glycolylneuraminic acid of tri-/tetraantennary glycans, (***H***) Galactosylated diantennary, (***I***) Core fucosylation (***J***) Antennary fucosylation. Data are shown as mean ± SEM of male (represented by triangles) and female (represented by circle), ApoE$^{-/-}$ (n=16) and ApoE$^{-/-}$/ASGR1$^{-/-}$ (n=14). Each symbol in the graph represents an individual value.

Supplement Figure S3

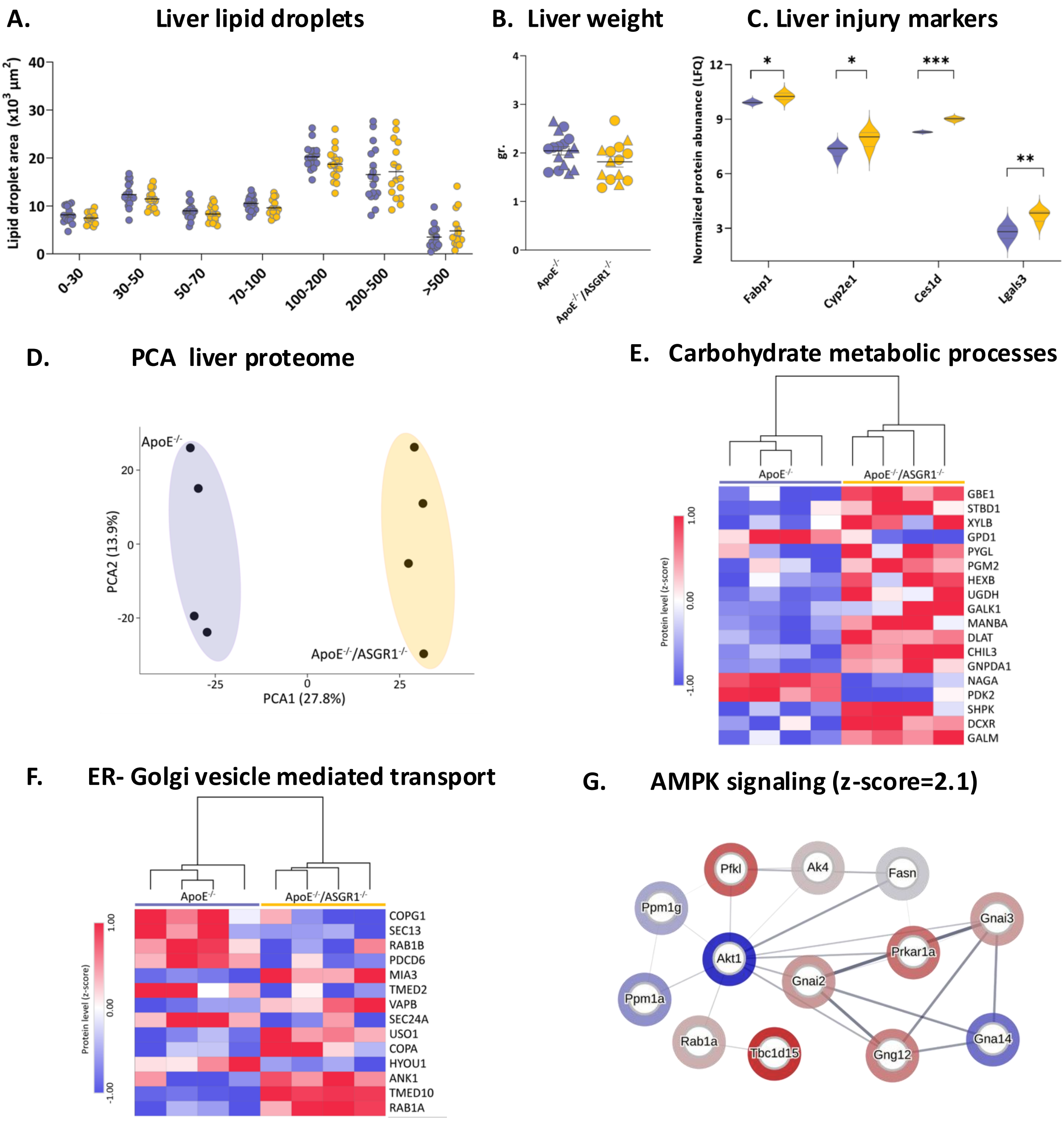

Supplementary Figure S3 – Liver function in ASGR1 deficiency in atherosclerotic mice. Liver was collected after 16 weeks of western type diet (WTD) in male (triangles) and female (circle) in ApoE$^{-/-}$ and ApoE$^{-/-}$/ASGR1$^{-/-}$ mice. (***A***) Liver lipid droplet area (expressed as µm^2), (***B***) Liver weight (gr.) in ApoE$^{-/-}$ (n=16) and ApoE$^{-/-}$/ASGR1$^{-/-}$ (n=14) mice, (***C-G***) Untargeted liver proteomics in ApoE$^{-/-}$ (n=4) and ApoE$^{-/-}$/ASGR1$^{-/-}$ (n=4) mice, (***C***) Liver injury markers, (***D***) Principal component analysis (PCA) of total liver proteome, Heatmaps with hierarchical clustering showing the protein levels for the gene ontology (GO) pathways on differently expressed proteins (DEPs, $p<0.05$) for (***E***) Carbohydrate metabolic processes (***F***) ER-Golgi vesicle mediated transport. Pathways with FDR<0.05 were consider for GO (***G***) Network analysis on proteins related with the activation of AMKP signaling pathway (z-score=2.1) based on the IPA analysis of the DEPs. The red and blue nodes present the up-, downregulated nodes, respectively.
